# Relationship between advanced glycation end-products, serum carnosinase-1 and diabetic nephropathy and diabetic retinopathy

**DOI:** 10.5937/jomb0-58488

**Published:** 2026-01-06

**Authors:** Yu Rong

**Affiliations:** 1 Tianjin Eye Hospital, Department of Clinical Laboratory, Tianjin, China

**Keywords:** AGEs, CN-1, DN, DR, AGEs, CN-1, DN, DR

## Abstract

**Background:**

This article analysed the relationship between serum advanced glycation end-products (AGEs), carnosinase-1 (CN-1) and diabetic nephropathy (DN) and diabetic retinopathy (DR).

**Methods:**

150 patients with type 2 diabetes mellitus (DM2) were grouped: DN and non-DN, DR and non-DR groups. Fasting venous blood was collected, and serum levels of AGEs and CN-1 were detected. Pearson's correlation (PC) test was adopted to analyse their correlation with DN and DR, and multivariate logistic regression (MLR) analysis was adopted.

**Results:**

There were 48 DN cases, 102 non-DN cases, 20 DR cases, and 130 non-DR cases in 150 patients with DM2. As against the non-DN group, the serum levels of AGEs and CN-1 in the subjects with DN were markedly increased. Similarly, the serum levels of AGEs and CN-1 in subjects with DR were also significantly increased compared to the non-DR group. The results of correlation analysis revealed that the levels of serum AGEs and CN-1 were positively correlated with the occurrence of DN and DR. Serum AGEs and CN-1 levels were identified as independent risk factors (IRF) for DN and DR (all P&lt; 0.05).

**Conclusions:**

AGEs and CN-1 may become new targets for the diagnosis and treatment of diabetic microvascular complications.

## Abstract (sr)

**Uvod:**

Ovaj članak analizirao je povezanost između serumskih produkata napredne glikacije (AGEs), karnozinaze-1 (CN-1) i dijabetičke nefropatije (DN) i dijabetičke retinopatije (DR).

**Metode:**

Ispitivanje je obuhvatilo 150 pacijenata sa dijabetesom tipa 2 (DM2), koji su grupisani u: DN i ne-DN grupu, kao i DR i ne-DR grupu. Prikupljeni su uzorci venske krvi na prazan stomak i određeni su nivoi AGEs i CN-1 u serumu. Za analizu korelacije sa DN i DR korišćen je Pirsonov test korelacije (PC), dok je za višestruku analizu primenjena multivarijantna logistička regresija (MLR).

**Rezultati:**

Među 150 pacijenata sa DM2, bilo je 48 slučajeva sa DN i 102 bez DN, kao i 20 slučajeva sa DR i 130 bez DR. U poređenju sa ne-DN grupom, nivoi AGEs i CN-1 u serumu kod ispitanika sa DN su bili značajno povećani. Slično tome, i kod ispitanika sa DR su nivoi AGEs i CN-1 u serumu bili znatno viši u odnosu na ne-DR grupu. Rezultati analize korelacije su pokazali da su nivoi AGEs i CN-1 pozitivno korelisali sa pojavom DN i DR. Nivoi AGEs i CN-1 u serumu identifikovani su kao nezavisni faktori rizika (IRF) za DN i DR (svi P&lt; 0,05).

**Zaključak:**

AGEs i CN-1 mogu da postanu nove stavke za dijagnostiku i lečenje mikrovaskularnih komplikacija dijabetesa.

## Introduction

More than 90% of diabetes mellitus (DM) patients are diagnosed with type 2 diabetes mellitus (DM2), which is characterised by hyperglycemia, insulin deficiency, and insulin resistance [1] [2]. Long-term hyperglycemia can lead to a variety of complications, including heart disease, retinopathy, renal failure, and more [3]. Diabetic nephropathy (DN) is a common chronic complication of DM, resulting from chronic kidney damage caused by prolonged hyperglycemia. Patients with DN exhibit progressive proteinuria, renal dysfunction, and increased blood pressure, which can ultimately threaten life [4]. Diabetic retinopathy (DR), a type of fundus disease in diabetic microangiopathy, is also highly prevalent, with significant rates of blindness and recurrence [5]. The pathogenesis of both DN and DR, however, remains incompletely understood.

Advanced glycation end-products (AGEs) are stable compounds formed by the non-enzymatic glycation of proteins, lipids, or nucleic acids. AGEs accumulate in tissues and can influence intracellular signalling and gene expression by binding to specific receptors on the cell membrane [6]. AGEs also trigger oxidative stress cascades, leading to tissue damage [7]. Consequently, AGEs have been implicated in the development of several diseases, including diabetes mellitus (DM), nephropathy, and atherosclerosis [8] [9] [10]. Carnosine, an endogenous dipeptide, is degraded by the enzyme carnosinase. It regulates protein glycation, potentially affecting the formation of AGEs [11]. Carnosinase-1 (CN-1) is the rate-limiting enzyme for carnosinase hydrolysis, but the role of CN-1 in diabetic microangiopathy, particularly in DR, is underexplored.

While advanced glycation end-products (AGEs) have been extensively studied in the context of diabetic nephropathy (DN), the involvement of CN-1 in diabetic retinopathy (DR) is relatively understudied. Although some research has shown the link between AGEs and DR, CN-1's contribution to the pathogenesis of DR remains speculative. This study aims to bridge this gap by exploring the relationship between serum CN-1 levels and DR in patients with DM2. By focusing on this underexplored area, we strive to contribute valuable insights into the role of CN-1 in diabetic microvascular complications, particularly DR.

This study aims to investigate the relationship between serum levels of AGEs and CN-1 in diabetic microvascular complications, specifically DN and DR in patients with DM2. We seek to explore how these biomarkers correlate with the occurrence of DN and DR, with an emphasis on the underexplored role of CN-1 in DR. Ultimately, the study aims to identify potential therapeutic targets for managing these complications.

## Materials and methods

### Study subjects

One hundred fifty subjects with DM2 in the Tianjin Eye Hospital from January to June 2024 were selected. The clinical data of subjects, gender, age, body mass index (BMI), basic medical history, duration of DM, systolic blood pressure (SBP), diastolic blood pressure (DBP), etc., were collected. Inclusion criteria: (1) patients met the diagnostic criteria of DM2 set by the WHO in 1999, with clinical symptoms of DM, fasting plasma glucose (FPG) 7.0 mmol/L or 2 h blood glucose (BG) after oral glucose tolerance test 11.1 mmol/L; (2) complete clinical data; (3) informed consent was obtained from patients. Exclusion criteria: (1) severe heart, liver, kidney and other essential organ diseases; (2) malignant tumors; (3) urinary tract infection or fever; (4) previous history of ocular trauma or ocular surgery; (5) autoimmune diseases; (6) mental diseases; (7) pregnant or lactating women; (8) renal disease caused by other causes. The trial obtained approval from the Tianjin Eye Hospital Ethics Committee(KY-2025034).

### Study design and participants

We included 150 patients with DM2, divided into two groups: DN and non-DN, DR and non-DR groups. For our analysis, we considered potential confounders such as medication use (e.g., ACE inhibitors, statins), smoking status, and dietary habits. These variables were adjusted for in the statistical models to ensure the robustness of our findings.

### Grouping method

It refers to the diagnosis of DN in the American DM Association and the American Kidney Disease Foundation [12]. Urinary albumin-to-creatinine (Cr) ratio (ACR) >30 mg/g or estimated glomerular filtration rate (eGFR) <60 mL/(minx1.73 m^2^) and symptoms lasting for more than 3 months were considered. The reference range of urinary ACR was <30 mg/g, microalbuminuria was defined as 30 urinary ACR <300 mg/g, and macroalbuminuria as urinary ACR 300 mg/g. There were DN and non-DN groups.

The diagnostic criteria of DR were referred to [13]. Retinal haemorrhage, hard/soft exudation, neovascularisation, and other diabetic characteristic changes were observed by ophthalmoscopy. Excluding the influence of different factors, the patient developed symptoms related to peripheral nerve dysfunction, and the conduction velocity of the median nerve, the ulnar nerve, the radial nerve, and the peroneal nerve was examined by electromyography. There were DR and non-DR groups.

### Detection of biochemical indicators

Blood routine: Fasting venous blood was collected, and the following parameters were detected: fasting plasma glucose (FPG), serum creatinine (Cr), triglycerides (TG), total cholesterol (TC), low-density lipoprotein cholesterol (LDL-C), high-density lipoprotein cholesterol (HDL-C), and glycated haemoglobin (HbA1c).

Urine routine indicators: the mid-morning urine of the patients was collected for the detection of urine albumin, Cr, and eGFR, and the ACR was calculated.

Serum CN-1: Fasting venous blood was subjected to centrifugation at 1000 r/min for 10 min without anticoagulant treatment. The content of CN-1 was detected according to the instructions of the ELISA kit. Blank, standard, and sample wells were set, and 100 μL of dilution, standard, and serum were added to each well in turn. Following capping, the mixture was incubated for 2 h at 37°C. Diluted biotin-labelled CN-1 antibody 100 μL was incubated for 1 h at 37°C after capping. 200 μL of washing solution was applied, and the plates were rinsed three times. 100 μL of diluted horseradish peroxidase-labelled antibody was applied for 1 h at 37°C after capping. Following rinsing the plates three times, 90 μL of substrate solution was used, 30 min at 37°C in the dark. The reaction was terminated by adding 50 μL termination solution, and the absorbance was detected at 450 nm. The serum CN-1 ELISA kit was purchased from Beijing Vicbio Biotechnology Co., Ltd.

Serum AGEs: The serum of patients was used as samples, and the content of AGEs was detected according to the operation steps in the kit. Blank, standard, and sample wells were set up. Except for the blank wells, 50 μL of standard products and serum were applied in turn, incubated for 30 min at 37°C after capping. 200 μL of washing solution was applied, and the plate was rinsed three times. Except for blank wells, 50 μL of enzyme-labelled reagent was used, incubated for 30 min at 37°C after capping. The plates were rinsed three times, and 50 μL of chromogenic solutions A and B were applied. After mixing, the plates were incubated at 37°C for 10 min. The reaction was terminated to detect the absorbance. Serum AGEs ELISA kit was purchased from Shanghai Yaji Biotechnology Co., LTD.

### Statistical methods

SPSS 23.0 was used for analysis. Qualitative data are presented as frequencies (%), with χ^2^ or Fisher's test. Quantitative data with normal distribution were described as mean ± SD and analysed using t-tests. Non-normally distributed data were presented as median (IQR) and analysed with the Mann-Whitney U test. PC and MLR analyses were performed, with adjustments for confounders (e.g., medication use, smoking, dietary habits). Results are reported with adjusted odds ratios (ORs) and 95% confidence intervals (CIs), with P<0.05 considered statistically significant.

## Results

### Contrast of data between subjects with and without dn

### (1) Contrast of general data

According to the diagnostic criteria, there were 48 cases with DN and 102 cases without DN. Gender, age, course of disease, BMI, SBP and DBP had no obvious distinction (*P*>0.05) (Table 1).

**Table 1:** Contrast of general data. Footnote: DN (Diabetic Nephropathy), BMI (Body Mass Index), SBP (Systolic Blood Pressure), DBP (Diastolic Blood Pressure).

Information	DN group<br>(n= 48)	Non-DN group<br>(n = 102)	* χ^2^ or t *	* P *
Gender [n(%)]			0.516	0.489
Male	25 (52.08)	56 (54.90)		
Female	23 (47.92)	46 (45.10)		
Age (years)	56.71±10.46	57.23±10.85	-1.559	0.174
Duration of illness (years)	8.95±1.77	9.08±1.85	-0.975	0.583
BMI (kg/m^2^)	24.18±2.05	24.37±2.11	-0.452	0.767
SBP (mmHg)	140.52±11.36	138.97±12.12	-0.884	0.610
DBP (mmHg)	78.09±6.67	77.86±6.80	-0.896	0.624

### (2) Contrast of biochemical indicators

**Figure 1:**
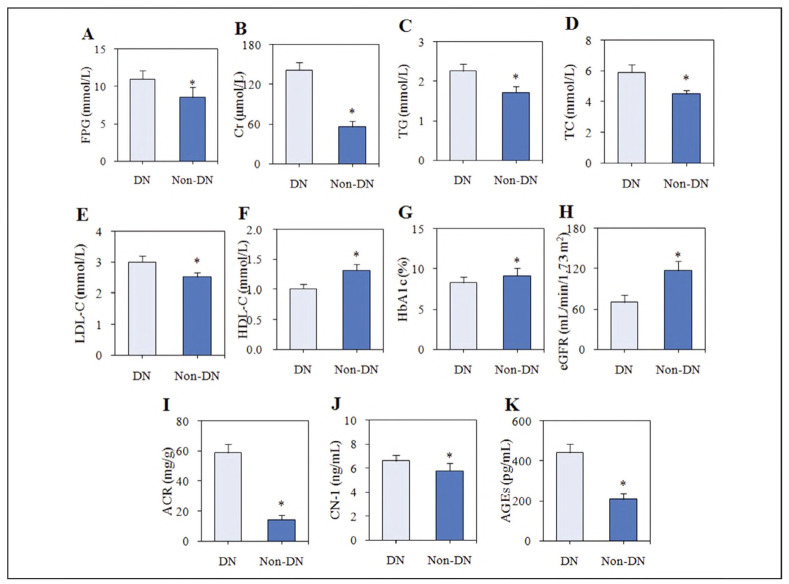
Contrast of the levels of biochemical indicators between patients with and without DN. Notes: (A) FPG; (B) serum Cr; (C) TG; (D) TC; (E) LDL-C; (F) HDL-C; (G) HbA1c;(H) eGFR; (I) ACR; (J) CN-1; (K) AGEs; As against subjects with DN, *P < 0.05

The levels of biochemical indexes in serum and urine were compared in subjects. Compared to non-DN subjects, HDL-C, HbA1c, and eGFR were significantly lower, while FPG, Cr, TG, TC, LDL-C, ACR, CN-1, and AGEs were significantly higher in subjects with DN (P<0.05) (Figure 1).

### Contrast of data between dr and non-dr subjects

### (1) Contrast of general data

According to the diagnostic criteria, there were 20 cases with DR and 130 cases without DR. There were no differences in gender, age, disease course, BMI, systolic blood pressure (SBP), and diastolic blood pressure (DBP) among subjects (P > 0.05) (Table 2).

**Table 2:** Contrast of general data. Footnote: DR (Diabetic Retinopathy), BMI (Body Mass Index), SBP (Systolic Blood Pressure), DBP (Diastolic Blood Pressure).

Information	Group DR (n = 20)	Non-DR group (n = 130)	* χ^2^ or t *	* P *
Gender [n(%)]			0.081	0.767
Male	11 (55.00)	70 (53.85)		
Female	9 (45.00)	60 (46.15)		
Age (years)	55.86±11.53	56.71±10.94	-0.719	0.516
Duration of illness (years)	8.53±1.60	8.62±1.53	-0.752	0.483
BMI (kg/m^2^)	24.25±2.17	24.30±2.08	-0.560	0.781
SBP (mmHg)	142.31±12.58	140.78±12.44	-0.092	0.975
DBP (mmHg)	78.23±7.19	78.09±6.96	-0.088	0.992

### (2) Contrast of biochemical indicators

**Figure 2:**
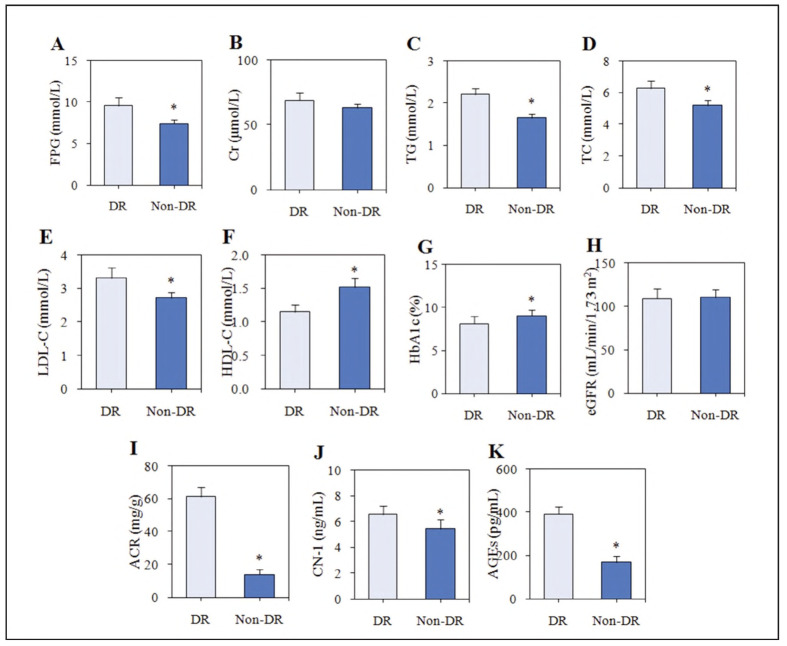
Contrast of the levels of biochemical indicators between patients with and without DR.

Cr and eGFR levels had no obvious distinction in the non-DR subjects and the DR subjects (*P*>0.05). As against the non-DR subjects, HDL-C and HbA1c were markedly lower, and fPg, Tg, TC, LDL-C, ACR, CN-1, and AGEs were markedly higher in the subjects with Dr (*P*<0.05) (Figure 2).

### Correlation analysis of each biochemical index with DN and DR

### (1) Correlation analysis with DN

**Figure 3:**
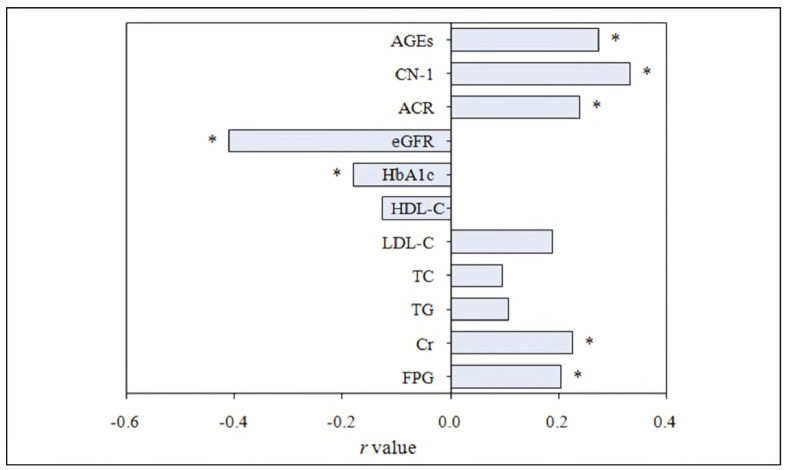
PC analysis between each biochemical index and DN. Note: * indicates obvious correlation, P < 0.05

PC test was adopted. FPG, Cr, ACR, CN-1, and AGEs were positively correlated with DN. In contrast, HbA1c and eGFR showed a marked negative correlation with DN (all P < 0.05) (Figure 3).

### Correlation analysis with DR

**Figure 4:**
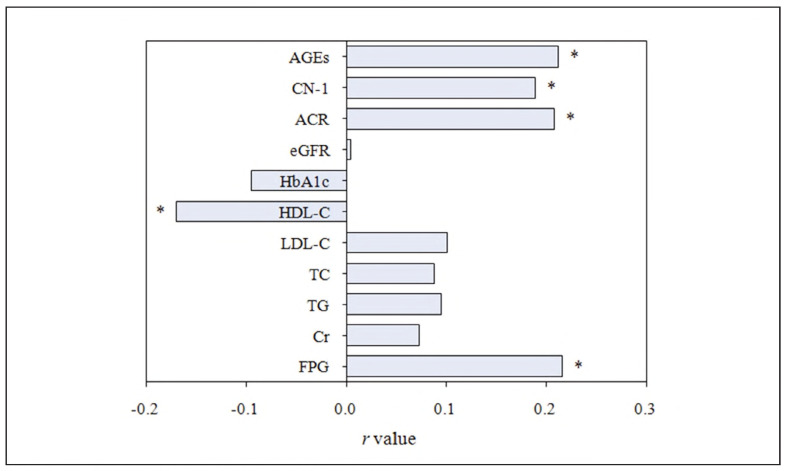
PC analysis between each biochemical index and DR. Note: * P < 0.05

PC test was adopted. FPG, ACR, CN-1, and AGEs showed a positive correlation with DR, while HDL-C exhibited a significant negative correlation with DR (all P<0.05) (Figure 4).

### Analysis of the influencing factors of DN and DR

### Analysis of the influencing factors of DN

MLR analysis was adopted. CN-1 and AGEs were IRF for DN, while HbA1c was an independent protective factor (all *P*<0.05) (Table 3).

**Table 3:** MLR analysis of DN. Footnote: FPG (Fasting Plasma Glucose), HbA1c (Glycated Haemoglobin), CN-1 (Carnosinase-1), AGEs (Advanced Glycation End-products).

Factors	*β*	* SE *	* OR *	* 95% CI *	* P *
FPG	0.562	0.157	0.906	0.121-1.318	0.123
HbA1c	-0.670	0.073	4.413	1.109-7.554	0.025
CN-1	1.037	0.190	2.174	0.553-6.782	0.001
AGEs	0.896	0.457	3.982	0.682-5.549	0.001

### Analysis of the influencing factors of DR

MLR analysis was adopted. CN-1 and AGEs were identified as independent risk factors for DR, while HDL-C was found to be a protective factor for DR (all P < 0.05) (Table 4).

**Table 4:** MLR analysis of DR. Footnote: FPG (Fasting Plasma Glucose), HDL-C (High-Density Lipoprotein Cholesterol), CN-1 (Carnosinase-1), AGEs (Advanced Glycation End-products).

Factors	*β*	* SE *	* OR *	* 95% CI *	* P *
FPG	0.685	0.480	1.251	0.228~3.371	0.156
HDL-C	-1.557	0.613	5.306	1.462~10.495	0.012
CN-1	1.269	0.445	3.250	1.096~5.736	0.001
AGEs	1.146	0.627	4.073	1.430~6.673	0.001

## Discussion

The findings of this study provide insight into the role of CN-1 in diabetic retinopathy, a topic with limited prior research. While much of the existing literature focuses on well-known biomarkers, such as AGEs, the specific involvement of CN-1 in diabetic retinopathy (DR) has not been thoroughly explored. This study highlights CN-1 as an independent risk factor for DR, demonstrating its potential as a novel therapeutic target for the management of diabetic microangiopathies. Given the scarcity of studies on CN-1 and its correlation with DR, our research contributes a unique perspective to the growing body of knowledge on diabetic complications.

DM2 is a kind of disease that causes a series of metabolic disorders, such as persistent hyperglycemia and pathological changes due to insulin resistance. As DM2 progresses, it can lead to a range of cardiovascular and cerebrovascular complications, such as diabetic retinopathy (DR), diabetic nephropathy (DN), and diabetic peripheral neuropathy [14]. This article analysed the relationship between AGEs and CN-1 levels and diabetic microangiopathy.

AGEs refer to stable compounds formed by the reaction of macromolecular substances such as lipids and proteins with carbonyl groups in reducing sugars in the non-enzymatic environment [15]. Under normal physiological conditions, the production rate of AGEs is slow and in a relatively balanced state. With the circulation of the body, AGEs can be degraded by liver cells and excreted from the body through the kidneys [16]. AGEs can be formed in the state of hyperglycemia, and AGEs can also be produced in the process of glucose auto-oxidation, oxidative stress and inflammatory response [17]. Intracellular and extracellular proteins are modified by sugar to produce AGEs. This process is irreversible, and the accumulation of AGEs will lead to the loss of protein function. Sustained high BG levels can promote the non-enzymatically catalysed glycosylation of proteins and glucose and the formation of AGEs [18]. The level of AGEs in the human body is related to BG concentration, and the level of AGEs in diabetic patients is often increased [19]. In this article, the serum levels of AGEs in DN subjects were markedly higher than those in non-DN subjects, and the serum levels of AGEs in DR subjects were also considerably higher than those in non-DR subjects. The increased levels of AGEs in patients with DM2 will increase the degree of islet cell dysfunction and insulin resistance, and eventually further aggravate hyperglycemia [20]. The formation and accumulation of AGEs are closely related to the process of diabetic macroangiopathy [21].

The incidence of DN is increasing year by year, and it is the primary factor leading to end-stage renal failure in patients. At present, it is believed that hyperglycemia, oxidative stress, inflammation, and glycosylation are all involved in the process of DN [22] [23] [24]. Fukami et al. [25] suggested that AGEs can participate in the process of DN by stimulating oxidative stress and inducing excessive production of various growth factors and cytokines. Kaida et al. [26] proposed that the levels of AGEs were abnormally increased in the glomeruli of the mouse model of type 2 DN, and blocking the AGEs-RAGE axis could prevent DN. In this article, serum AGEs are positively correlated with the occurrence of DN, and it is an IRF for the occurrence of DN. In addition, in this article, HbA1c is a protective factor for DN. Haemoglobin A1c is an essential indicator for evaluating blood glucose control. With the prolongation of the course of DM, the kidney is exposed to high glucose for a longer time, which will cause a decrease in insulin receptor sensitivity and islet function in the body, and increase the risk of DN [27].

DR is a common complication of diabetic microangiopathy. The main manifestations of DR are vascular proliferation, microaneurysms and haemorrhage, which are related to factors such as capillary basement membrane thickening and permeability enhancement [28]. Previous studies have confirmed that oxidative stress, polyol pathway activation, and AGEs formation are potential mechanisms leading to DR. Mishra et al. [29] proposed that AGEs can cause structural and functional damage to retinal pigment epithelial cells. According to Stirban et al. [30], the accumulation of AGEs can damage the normal physiological function of pericytes and vascular endothelial cells. Ai et al. [31] proposed that the accumulation of AGE precursors and AGEs occurs in the retinal microvascular wall of early DR, and AGEs can cause damage to retinal microvascular function. In this article, serum AGEs are positively correlated with the occurrence of DR, and is an IRF for the occurrence of DR. This is similar to the results of Ying et al. [32], who proposed that the content of AGEs and HbA1c in the skin have similar predictive value for DR and that AGEs can be used as a predictor of the incidence and poor prognosis of DN. This article shows that HDL-C is closely related to the occurrence of DR, which is a protective factor for DR.

We acknowledge that some studies have reported no significant association between advanced glycation end-products (AGEs) and microvascular complications, such as diabetic nephropathy (DN) and diabetic retinopathy (DR) [17] [33]. These findings suggest that the role of AGEs in microvascular complications may not be as prominent as previously thought. Furthermore, other research indicated that AGEs may have a stronger correlation with macrovascular diseases, such as cardiovascular disease, rather than microvascular conditions [34] [35]. These contradictory findings highlight the complexity of AGEs' role in diabetes-related complications and suggest that further investigation is necessary to clarify the specific mechanisms through which AGEs contribute to vascular damage in both macrovascular and microvascular contexts.

Serum CN-1 is a nonspecific dipeptidase in the cytosol, which plays a significant role in the process of some diseases, such as DM and kidney diseases. Qiu et al. [36] proposed that increased CN-1 levels are related to DN. Rodriguez-Nino et al. [37] suggested that DM mice exhibit nephritis and glomerular hypertrophy, etc. CN-1 can affect the intraglomerular pressure by regulating the tension of afferent glomerular arterioles, and is related to glomerular size, hypertrophy and Nephrin expression. Rodriguez-Nino et al. [37] proposed that urinary CN-1 levels were markedly increased in patients with DM2, and CN-1 excretion rates were higher in patients with severe renal impairment. In this article, serum CN-1 levels in subjects with DN were markedly higher than those in subjects without DN. Carnosine can improve insulin secretion and, finally, improve the state of hyperglycemia. Pfeffer et al. [38] proposed that the concentration of AGEs in DM-related kidneys was decreased, and the levels of interstitial inflammation and fibrosis were decreased in CN-1 knock-out mice. Peters et al. [39] proposed that CN has a potential angiotensin-converting enzyme inhibitor effect, which plays a role in reducing glomerular hyperfiltration rate by inhibiting the angiotensin-enzyme system and ultimately protecting renal function. In this article, serum CN-1 is positively correlated with the occurrence of DN, and it is an IRF for the occurrence of DN.

Ren et al. [40] proposed that CN exists in the retina, basal ganglia, and choroid plexus epithelial cells. However, the relationship between CN-1 and DR has not been fully elucidated. Serum CN-1 level in subjects with DR is also markedly higher than in subjects without DR, and the level of CN-1 is positively correlated with the occurrence of DR, which is an IRF for the occurrence of DR. The results indicate that CN-1 is closely related to the occurrence of DR, a finding that can be further verified in future studies.

## Conclusion

The levels of serum AGEs and CN-1 are closely related to the occurrence of diabetic microangiopathy (DN and DR). Elevated serum AGEs and CN-1 levels are IRF for diabetic microangiopathy. In the future, corresponding cell or animal models can be prepared to analyse the effect of AGEs or CN-1 expression on the progression of diabetic microangiopathy. In conclusion, AGEs and CN-1 may be potential therapeutic targets for diabetic microvascular complications such as DN and DR.

## Dodatak

### Conflict of interest statement

All the authors declare that they have no conflict of interest in this work.
